# LINKING VISUAL-SOMATOSENSORY INTEGRATION TO COGNITIVE AND MOTOR OUTCOMES IN AGING

**DOI:** 10.1093/geroni/igz038.1751

**Published:** 2019-11-08

**Authors:** Jeannette R Mahoney

**Affiliations:** Albert Einstein College of Medicine, Bronx, New York, United States

## Abstract

Ability to successfully integrate simultaneous information relayed across multiple sensory systems is an integral aspect of daily functioning. Unisensory impairments have been individually linked to slower gait, functional decline, and increased risks for falls in aging. Yet, research investigating age-related changes in multisensory integration (MSI) processes still remains relatively scarce. To date, there has been converging evidence for larger behavioral multisensory effects in older compared to younger adults; however, the question of whether larger effects are actually beneficial remains largely unanswered. Findings from our studies provide support for differential multisensory processing in aging, where decreased magnitude of visual-somatosensory integration was associated with worse balance, increased falls, and slower gait. Furthermore, we established a link between visual-somatosensory integration and cognition in aging. That is, magnitude of visual-somatosensory integration was largest in older adults with normal cognitive functioning, and presence of MCI/dementia significantly decreased magnitude of visual-somatosensory integration which in turn adversely impacted balance and gait performance. While the effect of MSI has been attributed to basic degenerative changes in neuronal architecture during the aging process, this speculative interpretation has yet to be formally tested. Future studies are clearly needed to establish the structural and functional correlates of MSI in aging, specifically visual-somatosensory integration, in order to further establish the link between differential multisensory effects with other important age-related clinical outcomes. Nevertheless, these studies stress the importance of successful MSI in aging, and highlight the need for multisensory based interventions that could potentially ameliorate disability.

